# Quality of life by dysmenorrhea severity in young and adult Japanese females: A web-based cross-sectional study

**DOI:** 10.1371/journal.pone.0283130

**Published:** 2023-03-16

**Authors:** Rami Mizuta, Noriaki Maeda, Tsubasa Tashiro, Yuta Suzuki, Sakura Oda, Makoto Komiya, Yukio Urabe

**Affiliations:** 1 Graduate School of Biomedical and Health Sciences, Hiroshima University, Hiroshima, Japan; 2 Department of Physical Therapy, Faculty of Rehabilitation, Kyusyu Nutrition Welfare University, Fukuoka, Japan; Nazarbayev University School of Medicine, KAZAKHSTAN

## Abstract

Dysmenorrhea is a monthly menstrual pain that can limit a woman’s quality of life (QOL). The relationship between dysmenorrhea severity and QOL has been reported in several countries; however, the results cannot be generalized because lifestyle and cultural background affect menstrual pain. This study sought to uncover whether 1) different factors, such as emotions and ways of coping with symptoms, vary with the severity of dysmenorrhea and 2) the severity of dysmenorrhea ultimately affects QOL in Japan. A web-based cross-sectional survey was sent to 1000 Japanese females aged 16–30 years. The respondents were divided into two groups: those without dysmenorrhea (n = 24) and those with dysmenorrhea (n = 471). The severity of dysmenorrhea was classified using the Numerical Rating Scale as either mild (1–3), moderate (4–7), or severe (8–10). In total, 156 respondents reported mild dysmenorrhea, 249 reported moderate dysmenorrhea, and 66 reported severe dysmenorrhea. QOL was measured using the 26-item World Health Organization Quality of Life scale. One-way ANOVA and Kruskal-Wallis tests were used to compare QOL across different levels of dysmenorrhea severity, depending on normality. Ultimately, significant differences in QOL scores (p<0.001) were observed based on dysmenorrhea severity, with respondents with severe dysmenorrhea reporting the lowest QOL scores. Meanwhile, significant differences were observed in the physical, psychological, and environmental subscales (p<0.001, p<0.001, p = 0.019) across respondents with different levels of dysmenorrhea severity; notably, respondents with severe dysmenorrhea demonstrated a negative spiral of chronic pain, which may significantly impact QOL, and, relatedly, a relatively low psychological QOL. This study is the first to show the relationship between dysmenorrhea severity and QOL in Japanese females, who are more likely to experience negative feelings during menstruation.

## Introduction

“Dysmenorrhea” is the term used to describe painful menstrual cramps of uterine origin; the condition is divided into primary dysmenorrhea and secondary dysmenorrhea based on the existence of organic disease [1]. Primary dysmenorrhea is defined as menstrual pain in the absence of pelvic disease, with the main symptom being lower abdominal pain [2]. The pain appears along with the onset of menstruation, is most painful in the first 24–36 hours, and lasts for 2–3 days [3]. A review of studies on the prevalence of dysmenorrhea covering nearly 20 countries reported that 70% of women suffer from dysmenorrhea, regardless of their country’s economic status [4]. In Japan, about 80% of females reportedly suffer from dysmenorrhea [5]. Although the severity of dysmenorrhea varies among individuals [6], dysmenorrhea remains one of the most important health problems worldwide since it is experienced by most young and adult females.

This study sought to uncover the impact of dysmenorrhea severity on females’ daily lives because its prevalence is high. However, little social movement exists to deal with this problem adequately. This study included young adult women between the ages of 15 and 29 years. The average age of onset of menstruation in Japan is around 12 years [7]. Considering that it takes six months to a year for stable ovulation to be established and up to two years to experience menstrual cramps [2], it made sense to set the lower age limit to 15 years, which is two to three years after menstruation usually starts. The upper age limit was set to 29 years. According to data from a previous Japanese survey of 4230 people aged 20–49 years—567 of whom visited a gynecologist due to dysmenorrhea—62.6% of respondents in their 20s had functional dysmenorrhea and 37.4% had secondary dysmenorrhea [8]. However, these proportions changed as age increased: among respondents in their 30s, 46.4% had primary dysmenorrhea and 53.6% had secondary dysmenorrhea. This trend was also similar among women in their 40s. This study focused on primary dysmenorrhea; accordingly, it did not include patients over 30 years old to reduce the risk of including participants with secondary dysmenorrhea. Some authors have indicated that the typical age of onset of dysmenorrhea is 25 years or younger [9]. In addition, one study has shown that the severity of primary dysmenorrhea does not significantly decrease after 25 years [10]. Therefore, this study included respondents up to 29 years old.

Previous studies have shown that dysmenorrhea leads to poor academic performance and work efficiency [11, 12]. Moreover, studies have shown that it can reduce quality of life (QOL) [13, 14], which the World Health Organization (WHO) defines as “An individual’s perception of their position in the life in the context of the culture in which they live and in relation to their goals, expectations, standards and concerns” [15]. The term “health-related QOL” refers to the health aspect of QOL, which is generally thought to reflect the impact of illness and treatment on disability and daily functioning [16]. Considering these definitions, dysmenorrhea may not only interfere with health-related QOL due to the physical symptom of menstrual pain but may also interfere with overall QOL. Additionally, a survey that calculated labor losses, hospital visits, and medication costs due to dysmenorrhea estimated the annual socioeconomic loss in Japan to be 682.8 billion yen [17]. Although there is a movement to encourage women to join the workforce in many countries around the world, including Japan [18], dysmenorrhea may be an obstacle to this movement [11, 12]. Thus, investigating the impact of dysmenorrhea and severity of symptoms is important, not only to improve women’s health, but also to ensure their QOL. However, such work must be attentive to the fact that the relationship between dysmenorrhea and QOL cannot be generalized across countries [13]—cultural and social factors are closely related to dysmenorrhea; for example, the impact of dysmenorrhea on academic and recreational activities varies across groups of different ethnicities [19]. In the context of Japan, an existing survey study comparing Japan and Taiwan reported a lack of menstrual education, both at home and in the broader society, and, relatedly, a lack of information sharing about menstruation between parents and children in Japan [20]. This study suggests that menstruation is relatively tabooed in Japan; importantly, this may increase the impact of dysmenorrhea in social life. However, the relationship between dysmenorrhea and QOL in Japan has not yet been clarified.

In response to these gaps in the literature, this study had two main aims: 1) to compare various factors, such as emotions and methods of coping with symptoms, by dysmenorrhea severity and 2) to explore whether dysmenorrhea severity affects the QOL of young and adult females. The study’s hypothesis was that more severe dysmenorrhea is associated with a lower QOL. Significantly, the findings will provide basic information on QOL and other aspects of dysmenorrhea among Japanese women based on the severity of the disease, which may importantly emphasize the importance of coping with dysmenorrhea.

## Methods

### Study design

This study’s anonymous online survey was conducted from May 17, 2021, to July 7, 2021, with Google Forms (Alphabet, Mountain View, CA, USA). The participants were young and adult females aged 15–29 years old—the age group most likely to experience primary dysmenorrhea—living across Japan [2, 7, 8]. Twelve university faculty members, three high school teachers, and ten companies were asked to distribute the online survey to females after receiving permission from their organizations. The first page of the survey stated that the respondents should not answer the survey more than once by themselves and that they could respond anonymously. All data were fully anonymized before we accessed them. On the same page, the purpose and methods of the online survey were explained, and willingness to participate was obtained by clicking a button confirming participation in the online survey. Only respondents willing to participate in the investigation were allowed to proceed to the next page. Participation for minors was accompanied by a parental opt-out document, which stated that the parent or legal guardian could refuse to allow the minor to participate. Minors were not allowed to participate in the survey unless they clicked two buttons indicating that they themselves wished to participate in the study and that their parent or guardian had seen the opt-out document and allowed them to participate. The study was conducted in accordance with the recommendations of the Internet E-Survey Checklist for Reporting Results [21] and guidelines of the Declaration of Helsinki and its modified version and was approved by the Hiroshima University Epidemiology Ethics Committee (E-2431). In this study, no written or verbal consent was obtained because the questionnaires were completely anonymous. The consent to participate on the online form was reviewed and approved by this ethics committee. The inclusion criteria were as follows: 1) young and adult Japanese females aged 15–29 years, 2) residing in Japan during the survey period, 3) without a severe condition such as heart or malignant disease, and 4) agreeing to participate in this study. The exclusion criterion was a current or previous history of gynecological or psychiatric disorders. Participants self-reported their disease histories on their questionnaires. Gynecological disorders included cervical cancer, ovarian tumors, endometriosis, uterine fibroids, polycystic ovarian syndrome, and other conditions that can affect menstruation. As an ethical consideration, we were required to provide a "do not want to answer" option for some of the questions. The following efforts were made to reduce bias in this study: 1) The survey was conducted at multiple sites (12 universities, 3 high schools, and 10 companies) rather than at a single site to avoid selection bias; 2) participant information was anonymized; 3) because it was a web-based questionnaire, the researcher and participants were not acquainted with each other; and 4) the participants were grouped according to the results of the questionnaire, and they did not know which group they would belong to when they responded to the questionnaire.

### Survey items and data collection

#### Sociodemographic data and basic menstrual information

The questionnaire included basic information, such as current and previous history of gynecological and psychiatric disorders; history of serious illnesses, such as heart disease or cancer; age, weight, height, and body mass index (BMI), which was calculated from weight and height (kg/m^2^); menarche age; duration of pain; amount of menstruation [22]; menstrual cycle [23]; family history of dysmenorrhea; and physical activity level. The duration of pain was included in this study only if it was less than three days because prolonged pain increases the risk of secondary dysmenorrhea [3]. We classified the amount of menstruation into three levels according to the number of sanitary napkins used per day, based on a previous study [22]. The three levels are small (≤5 pads/day), moderate (5–7 pads/day), and large (≥7 pads/day); these levels were self-reported by the participants. To assess the amount of physical activity, the International Physical Activity Questionnaire–Short Form [24, 25] was used. Total physical activity was calculated using the weekly average amount of walking, moderate physical activity, and vigorous physical activity (metabolic equivalent tasks [METs]*mins/week). One MET is approximately equal to the energy consumption required for a person to sit quietly. The amount of physical activity was also classified into three levels: low (<600 METs*mins/week), moderate (600–2999 METs*mins/week), and high (≥3000 METs*mins/week) [26].

#### Lifestyle

We asked participants about the following six items related to lifestyle: smoking (presence/ absence), alcohol intake (high/low: consumption of ≥5/ <5 alcoholic beverages), sleep (≥7 hours/< 7 hours), breakfast (eating/not eating), eating between meals (eating/not eating), and exercise (≥2 times, 30 minutes/ a week; < 2 times, 30 minutes/ a week) [27, 28].

#### Intensity of pain associated with menstruation

In this study, “dysmenorrhea” was defined in accordance with a review conducted by the WHO, which delineated it as menstrual pain experienced within the past three months [29, 30]. The Numerical Rating Scale (NRS) was used to evaluate the intensity of pain experienced in the last three months or less during menstruation. The NRS is a 10-point scale, with higher scores indicating stronger pain. We classified scores of 1 to 3 as mild dysmenorrhea, scores of 4 to 7 as moderate dysmenorrhea, and scores of 8 to 10 as severe dysmenorrhea [14, 31]. A supplementary evaluation of menstrual pain, the short-form McGill Pain Questionnaire (SF-MPQ), was also used [32, 33]. The SF-MPQ is useful for analyzing both qualitative and quantitative aspects of pain and consists of 15 pain descriptions, 11 sensory and 4 affective. Each question was rated on a 0- to 3-point Likert scale, with higher scores indicating stronger levels of pain.

#### Negative emotions and well-being

The Menstrual Distress Questionnaire (MDQ) is a commonly used measurement scale to assess the severity of symptoms related to menstruation [34]. We used the subscale of “negative affect” to evaluate the negative psychological aspects of premenstrual, menstrual, and postmenstrual periods. Responses were scored using a 6-point Likert scale, ranging from 1 (no reaction at all) to 6 (acute or partially disabling), with higher scores indicating more severe mental symptoms.

To assess well-being and mental health, the Japanese version of the WHO Five Well-being Index (WHO-5-J) was used [35]. It consists of a total of five questions. Responses to each item are rated on a 6-point Likert scale from 0 to 5. The maximum score is 25 points, with higher scores indicating better well-being.

#### Coping strategies for symptoms of dysmenorrheal

Respondents were asked how they had coped with menstrual pain in the past three months. Respondents were allowed to respond with more than one coping strategy. The coping strategies included using painkillers or oral contraceptives, seeking medical consultations, consuming hot drinks, stretching, massaging, warming the body, and exercising. The listed strategies for coping in this study were based on a previous systematic review [36].

**QOL.** The Japanese version of the WHO/QOL-26 was used to assess QOL. This questionnaire consists of four domains: physical (7 items), psychological (6 items), social (3 items), and environmental (8 items) [37–39], and two other independent questions. Each questionnaire item was standardized into a 5-point ordinal scale, from 1 (worst) to 5 (best).

### Statistical analysis

Respondents were divided into mild, moderate, and severe dysmenorrhea groups depending on the intensity of menstrual pain. As there are no reports on the relationship between the degree of dysmenorrhea and QOL in Japanese women, the effect size expected when comparing the main outcome—QOL—across the three groups was not known. Therefore, we calculated the sample size using an effect size of 0.25, an alpha error of 0.05, and a mean power of 0.80 using G*power (version 3.1.9.2, Heinrich-Heine-University Düsseldorf, Düsseldorf, Germany) [40]. From these calculations, we estimated that a total of 159 respondents were required for the three groups, with 53 respondents per group. A descriptive analysis was performed, with quantitative variables expressed as means and standard deviations and qualitative variables examined as frequencies and percentages. The one-way ANOVA and Bonferroni post-hoc tests were used to compare height, well-being, and QOL (physical, psychological, and environmental) between the three groups after the Shapiro-Wilk test confirmed normality. The Kruskal-Wallis and Bonferroni post-hoc tests were conducted to compare other continuous variables scores, such as sociodemographic data, menstrual information (menarche age, duration of pain), the intensity of pain associated with menstruation, negative emotion, and reduced sense of well-being and QOL. A chi-square test was used to compare the distribution of basic menstrual information (amount of menstruation, menstrual cycle, family history of dysmenorrhea), lifestyle, and coping strategies across the three groups. All data were analyzed using IBM SPSS Statistics for Windows (version 23.0; IBM Corp., Armonk, NY, USA). The level of significance was set at 0.05.

## Results

**Fig 1:**
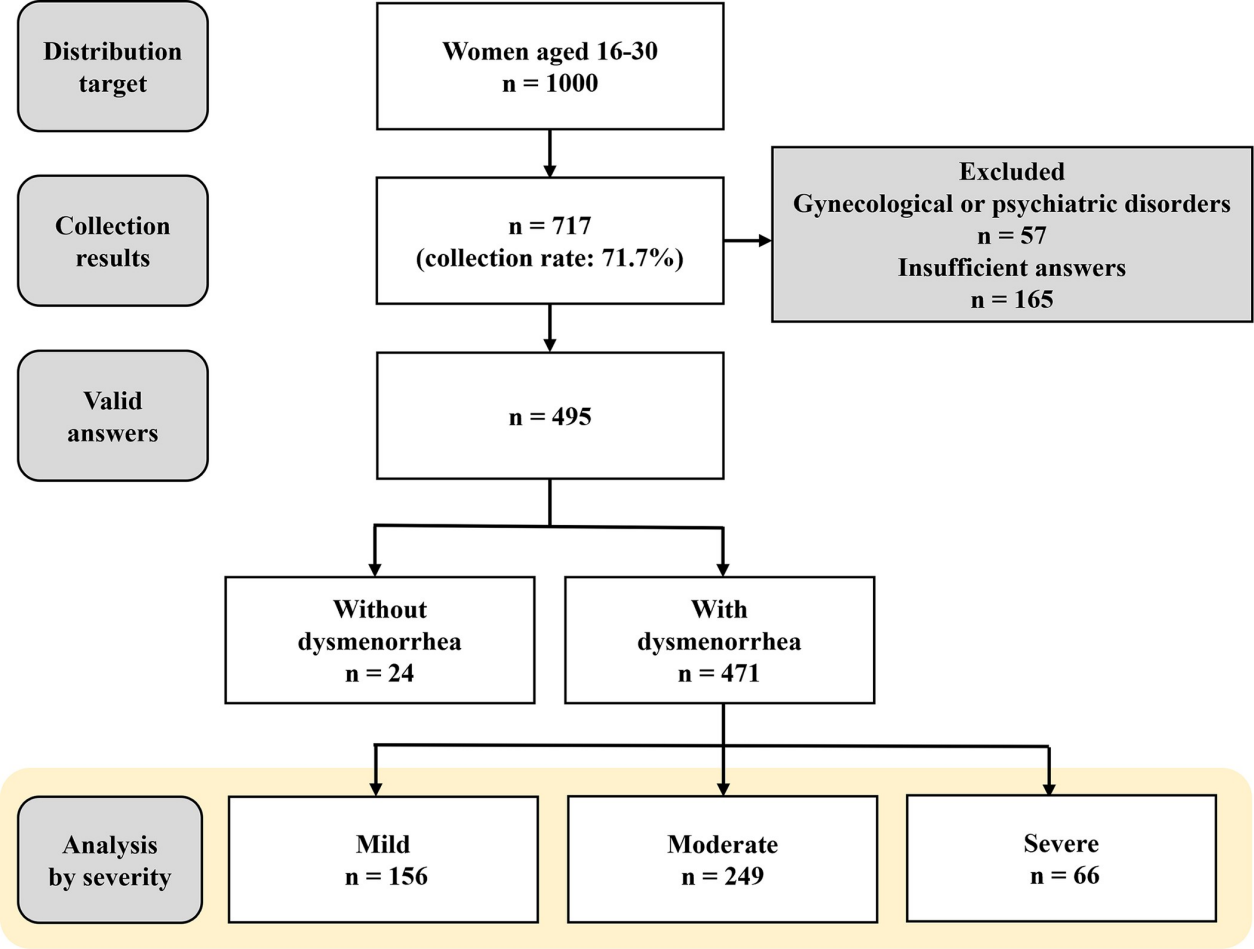
The flowchart of respondents and prevalence of dysmenorrhea in this study.

Of the 1000 distribution targets, we received answers from a total of 717 respondents (71.7%). Of these respondents, 57 had a current and previous history of gynecological or psychiatric disorders, and 165 responses were incomplete (with some respondents unwilling to answer certain questions). Therefore, 495 respondents (49.5%) were included in the current analysis. First, respondents were divided into two groups—without dysmenorrhea (n = 24) and with dysmenorrhea (n = 471)—depending on their responses to the questions on menstrual pain. Furthermore, as detailed above, the respondents with dysmenorrhea were classified into mild (n = 156), moderate (n = 249), and severe (n = 66) groups (Fig 1).

### Sociodemographic data and basic menstrual information of the mild, moderate, and severe groups

Table 1 shows the basic information and basic menstrual data of the dysmenorrhea respondents. The median age was 22.0 years in all groups. There were statistically significant differences in the duration of pain, amount of menstruation, and family history of dysmenorrhea across the three groups (p<0.05).

**Table 1:** Sociodemographic data and basic menstrual information of dysmenorrhea respondents.

Variables	Total	Mild	Moderate	Severe	p-value
	(n = 471)	(n = 156)	(n = 249)	(n = 66)	
**Age (years)**	22 (21.0–24.0)	22.0 (20.0–23.3)	22.0 (21.0–25.0)	22.0 (21.0–24.0)	0.026*
**Weight (kg)**	51.8 ± 6.1	51.8 ± 6.0	51.9 ± 6.1	51.1 ± 6.1	0.212
**Height (cm)**	158.7 ± 5.3	158.5 ± 5.0	158.8 ± 5.3	158.0 ± 5.4	0.218
**BMI (kg/m^2^)**	20.6 ± 2.1	20.6 ± 2.1	20.6 ± 2.1	20.5 ± 2.2	0.908
**Menarche age (years)**	12.4 ± 1.5	12.6 ± 1.5	12.3 ± 1.5	12.2 ± 1.5	0.063
**Duration of pain (days)**	1.5 ± 0.7	1.0 ± 0.6	1.6 ± 0.7	2.0 ± 0.7	<0.001*
**Amount of menstruation**					<0.001*
Small	113 (24.0)	58 (37.2)	48 (19.3)	7 (10.6)	
Moderate	315 (66.9)	90 (57.7)	183 (73.5)	42 (63.6)	
Large	43 (9.1)	8 (5.1)	18 (7.2)	17 (25.8)	
**Menstrual cycle**					0.552
Normal (25–38 days)	406 (86.2)	136 (87.2)	216 (86.7)	54 (81.8)	
Others (< 25 or > 38 days)	65 (13.8)	20 (12.8)	33 (13.3)	12 (18.2)	
**Family history of dysmenorrhea**					0.019*
Yes	153 (32.5)	37 (23.7)	86 (34.5)	30 (45.5)	
No	114 (24.2)	46 (29.5)	56 (22.5)	12 (18.2)	
Unknown	204 (43.3)	73 (46.8)	107 (43.0)	24 (36.3)	
**Physical activity level**					0.449
Low	156 (33.1)	44 (28.2)	90 (36.1)	22 (33.3)	
Moderate	200 (42.5)	69 (44.2)	105 (42.1)	26 (39.4)	
High	115 (24.4)	43 (27.6)	54 (21.7)	18 (27.3)	

Data are expressed as means ± standard deviation (SD) or n (%) or presented as medians and interquartile.

*: statistically significant. Post-hoc analysis for Age: Mild vs Moderate, p = 0.022; Mild vs Severe, p = 1.000; and Moderate vs Severe, p = 0.787. Post-hoc analysis for Duration of pain: Mild vs Moderate, p<0.001; Mild vs Severe, p<0.001; and Moderate vs Severe, p<0.001.

### Comparison of lifestyle of respondents classified by severity of dysmenorrhea

Table 2 presents the results of a comparison of information on lifestyle across the three groups. There were no statistically significant differences across the three groups in terms of smoking, alcohol consumption, sleeping, breakfast, eating between meals, and exercise (p>0.05).

**Table 2:** Comparison of lifestyle of respondents classified by severity of dysmenorrhea.

Variables	Total	Mild	Moderate	Severe	p-value
	(n = 471)	(n = 156)	(n = 249)	(n = 66)	
**Smoking**					0.634
Presence	28 (5.9)	7 (4.5)	16 (6.4)	5 (7.6)	
Absence	443 (94.1)	149 (95.5)	233 (93.6)	61 (92.4)	
**Alcohol consumption**					0.151
High	23 (4.9)	9 (5.8)	14 (5.6)	0 (0.0)	
Low	448 (95.1)	147 (94.2)	235 (95.5)	66 (100.0)	
**Sleeping**					0.387
≥7 hours	220 (46.7)	70 (44.9)	114 (45.8)	36 (54.5)	
< 7 hours	251 (53.3)	86 (55.1)	135 (54.2)	30 (45.5)	
**Breakfast**					0.059
Eating	358 (76.0)	125 (80.1)	190 (76.3)	43 (65.2)	
Not eating	113 (24.0)	31 (19.9)	59 (23.7)	23 (34.8)	
**Eating between meals**					0.565
Eating	329 (69.9)	104 (66.7)	177 (71.1)	48 (72.7)	
Not eating	142 (30.1)	52 (33.3)	72 (28.9)	18 (27.3)	
**Exercise**					0.217
≥2 times, 30 minutes/a week	215 (45.6)	75 (48.1)	105 (42.3)	35 (53.0)	
<2 times, 30 minutes/a week	256 (54.4)	81 (51.9)	144 (57.8)	31 (47.0)	

Data are expressed as n (%)

### Comparison of intensity of pain associated with menstruation and negative emotion of respondents with dysmenorrhea in the mild, moderate, and severe groups

Table 3 details the intensity of pain and negative emotion of respondents with dysmenorrhea. The NRS scores used to evaluate the intensity of menstrual pain were significantly different across the three groups (p<0.001). The SF-MPQ total, sensory, and affective scores on the supplemental assessment of pain were also significantly higher for the severe dysmenorrhea group (p<0.001, respectively). In terms of negative affect on the MDQ, the severe group showed higher scores and more negative emotions across periods (p<0.05). Well-being scores were also significantly lower in the severe group (p = 0.011).

**Table 3:** More information on intensity of pain and negative emotion of respondents with dysmenorrhea.

Variables	Total	Mild	Moderate	Severe	p-value	p-value mild vs moderate	p-value mild vs severe	p-value moderate vs severe
	(n = 471)	(n = 156)	(n = 249)	(n = 66)				
**NRS**	4.7 ± 2.5	2.2 ± 0.8	5.8 ± 1.1	8.3 ± 0.6	<0.001*	<0.001*	<0.001*	<0.001*
**SF-MPQ**	10.1 ± 8.1	3.4 ± 2.9	11.6 ± 6.9	20.2 ± 7.5	<0.001*	<0.001*	<0.001*	<0.001*
**total**								
**SF-MPQ**	7.3 ± 5.6	2.8 ± 2.3	8.4 ± 4.8	13.8 ± 5.4	<0.001*	<0.001*	<0.001*	<0.001*
**sensory**								
**SF-MPQ affective**	2.8 ± 3.0	0.6 ± 1.0	3.2 ± 2.7	6.4 ± 3.2	<0.001*	<0.001*	<0.001*	<0.001*
**MDQ**	14.3 ± 8.0	11.2 ± 4.9	15.1 ± 7.7	18.7 ± 11.6	<0.001*	<0.001*	<0.001*	0.326
**negative affect**								
**(pre-menstruation)**								
**MDQ**	14.2 ± 7.9	10.9 ± 4.9	14.7 ± 7.3	20.0 ± 11.6	<0.001*	<0.001*	<0.001*	0.004*
**negative affect**								
**(during menstruation)**								
**MDQ**	9.7 ± 4.6	8.7 ± 2.2	10.0 ± 4.6	11.3 ± 7.3	0.003*	0.008*	0.024*	1.000
**negative affect**								
**(post-menstruation)**								
**Well-being**	15.8 ± 4.8	16.5 ± 4.6	15.7 ± 4.9	14.6 ± 4.6	0.011*	0.266	0.009*	0.180

Data are expressed as means ± SD.

*: statistically significant. NRS: Numerical Rating Scale. SF-MPQ: Short-Form McGill Pain Questionnaire. MDQ: Menstrual Distress Questionnaire.

### Comparison of coping strategies for symptoms of dysmenorrhea with different levels of severity

Table 4 shows the comparison of coping strategies for the three groups classified by dysmenorrhea severity. The percentage of participants taking analgesics and oral contraceptives as a pharmacological measure was significantly higher as the severity of dysmenorrhea increased (p<0.001). Similarly, the percentage of respondents who had sought medical consultations was higher in the severe dysmenorrhea group (p<0.001), with 21.2% receiving medical consultations. There were no significant differences in the percentage of respondents coping by stretching or exercising across the three groups.

**Table 4:** Comparison of coping strategies for symptoms of dysmenorrhea across different levels of severity.

Variables	Total	Mild	Moderate	Severe	p-value
	(n = 471)	(n = 156)	(n = 249)	(n = 66)	
**Analgesics**					<0.001*
No	309 (65.6)	151 (96.8)	141 (56.6)	17 (25.8)	
Yes	162 (34.4)	5 (3.2)	108 (43.4)	49 (74.2)	
**Oral contraceptives**					<0.001*
No	445 (94.5)	153 (98.1)	238 (95.6)	54 (81.8)	
Yes	26 (5.5)	3 (1.9)	11 (4.4)	12 (18.2)	
**Medical consultation**					<0.001*
No	434 (92.1)	156 (100.0)	226 (90.8)	52 (78.8)	
Yes	37 (7.9)	0 (0.0)	23 (9.2)	14 (21.2)	
**Consuming a hot drink**					<0.001*
No	299 (63.5)	118 (75.6)	149 (59.8)	32 (48.5)	
Yes	172 (36.5)	38 (24.4)	100 (40.2)	34 (51.5)	
**Stretching**					0.331
No	430 (91.3)	144 (92.3)	229 (92.0)	57 (86.4)	
Yes	41 (8.7)	12 (7.7)	20 (8.0)	9 (13.6)	
**Massaging**					0.012*
No	422 (89.6)	146 (93.6)	223 (89.6)	53 (80.3)	
Yes	49 (10.4)	10 (6.4)	26 (10.4)	13 (19.7)	
**Warming the body**					<0.001*
No	316 (67.1)	128 (82.1)	155 (62.2)	33 (50.0)	
Yes	155 (32.9)	28 (17.9)	94 (37.8)	33 (50.0)	
**Exercising**					0.332
No	462 (98.1)	155 (99.4)	242 (97.2)	65 (98.5)	
Yes	9 (1.9)	1 (0.6)	7 (2.8)	1 (1.5)	

Data are expressed as n (%).

*: statistically significant.

### Comparison of QOL scores among mild, moderate, and severe dysmenorrhea groups

**Fig 2:**
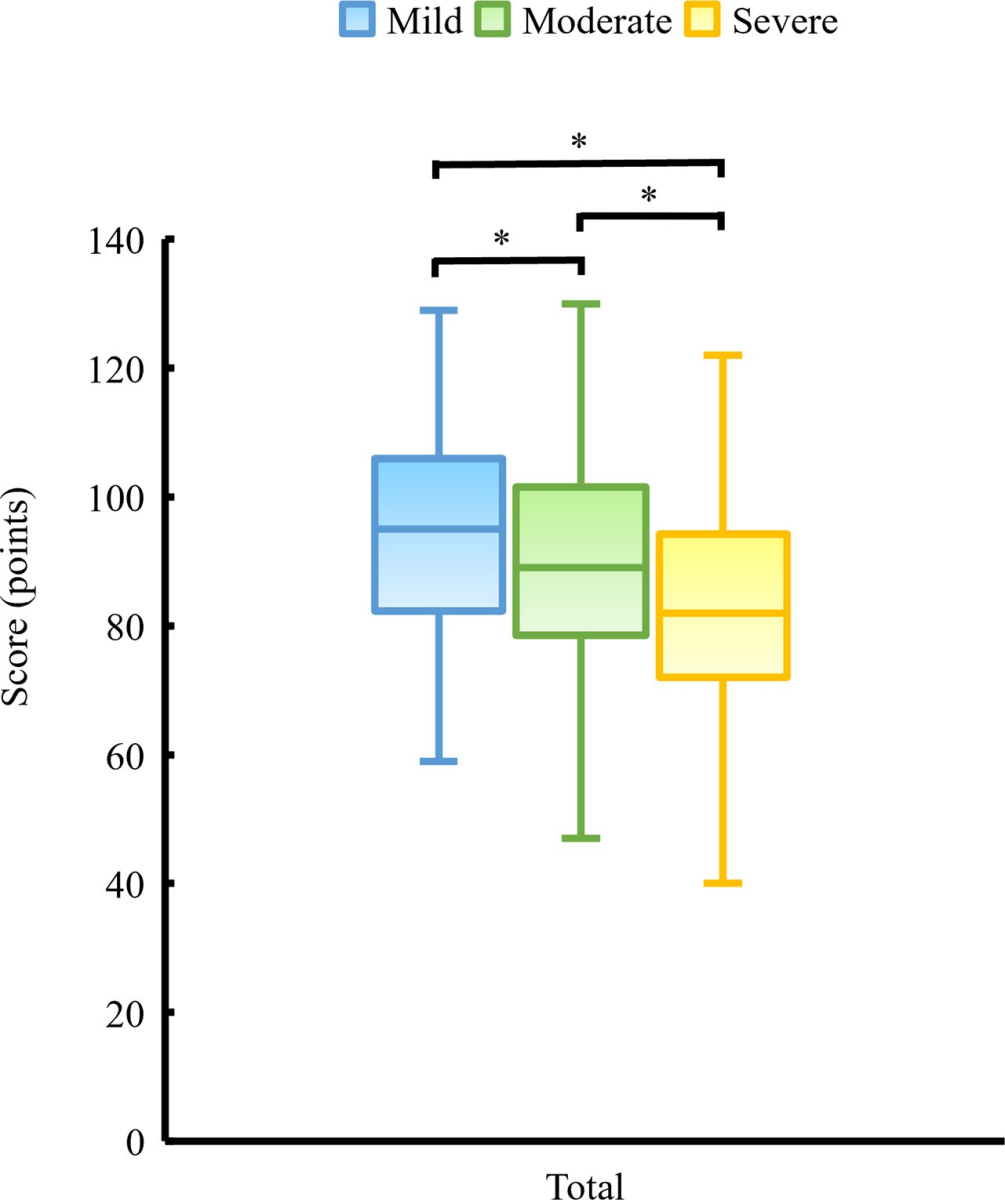
Comparison of the total QOL score of respondents classified by severity of dysmenorrhea. *Note*: *:statistically significant.

**Fig 3:**
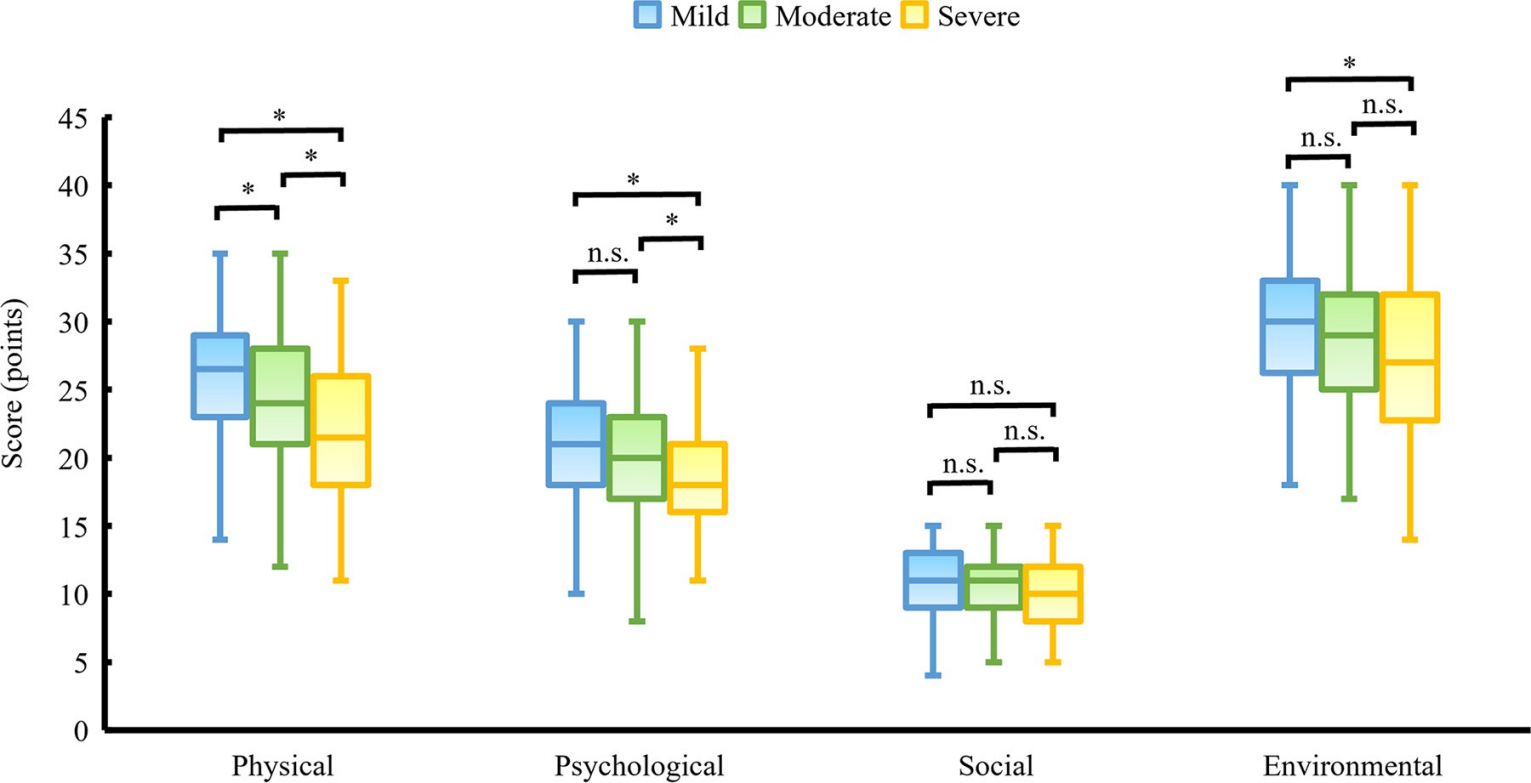
Comparison of the subscales of QOL scores of respondents classified by severity of dysmenorrhea. *Note*: *:statistically significant; n.s.: non-significant.

Fig 2 shows the comparison of the total QOL scores of respondents classified by the severity of dysmenorrhea. The mean total QOL scores of the mild, moderate, and severe groups were 93.9 points (SD = 16.4), 89.3 points (SD = 15.7), and 83.4 points (SD = 16.8), respectively (p<0.001, mild vs moderate: p = 0.015, mild vs severe: p<0.001, moderate vs severe: p = 0.022). Regarding the subscales, there were significant differences in physical, psychological, and environment QOL scores when the three groups were compared (p<0.001, p<0.001, p = 0.019, respectively), and severe dysmenorrhea group had the lowest QOL scores (Fig 3). The mean physical QOL scores were as follows: 25.9 points (SD = 4.9) in the mild group, 24.3 points (SD = 5.0) in the moderate group, and 22.0 points (SD = 4.8) in the severe group. The mean psychological QOL scores were 21.0 points (SD = 4.6) in the mild group, 20.1 (SD = 4.4) in the moderate group, and 18.1 (SD = 4.5) in the severe group. The mean environmental QOL scores were 29.7 points (SD = 5.4) in the mild group, 28.5 points (SD = 5.4) in the moderate group, and 27.6 points (SD = 6.2) in the severe group. The social QOL scores did not differ significantly across the three groups (p = 0.087, mild: 10.8±2.5 points, moderate: 10.5±2.4 points, severe: 10.0±3.0 points).

## Discussion

This study aimed to explore whether the severity of dysmenorrhea affects the QOL of young and adult females in Japan. Clarifying this relationship can lead to the development of measures to help those who may be experiencing difficulties in their social daily lives due to menstrual cramps. The results revealed that dysmenorrhea severity is related to differences in QOL: significant differences in QOL scores were observed across participants with mild, moderate, and severe dysmenorrhea, with the severe dysmenorrhea group demonstrating the lowest total QOL scores and lowest physical, psychological, and environmental QOL subscale scores. This is the first study to show that severe dysmenorrhea is related to a lower QOL in Japanese females. This important finding adds to studies conducted in other countries that show a correlation between severe dysmenorrhea and low QOL by confirming that a similar relationship exists in Japan. We were also able to add data that could be used to examine how negative emotions and psychogenic pain, which are easily felt by the Japanese, are related to the decline in QOL.

As a premise, we will compare the results of this study with those of previous studies on dysmenorrhea and QOL in other countries. A study conducted in China, a neighbor of Japan, similarly showed that dysmenorrhea severity is associated with lower health-related QOL. However, statistically significant differences across different levels of dysmenorrhea severity were only found for the QOL item of body pain, and no significant differences were observed in other areas, such as mental health [14]. Even though the current study used different QOL-related questionnaires, the results notably revealed that severe dysmenorrhea is related to a lower physical and environmental QOL. Meanwhile, the results of this study can also be compared with the findings of a study conducted in Spain [13], which is considered a high-income country like Japan [41]. In this Spanish study, although there was a tendency for overall QOL to decline as dysmenorrhea became more severe, there was no significant difference between the groups. This was also not the result for pain, and all other indicators, including mental health, were not significantly different. Considering these results, the severity of dysmenorrhea may be more likely to affect QOL in Japan.

The physical aspects of the WHO/QOL-26 included items that asked about the degree to which physical pain or discomfort limits daily activities. Previous studies in other countries have shown that dysmenorrhea affects performance at school and work [11, 12]. Importantly, we found that more severe dysmenorrhea was related to poorer performance. The environmental QOL domain included questions on topics such as whether the respondent felt they were living in a healthy environment and whether they were satisfied with the accessibility of healthcare facilities and social services. Only 21.2% of the severe group in this study had seen a medical provider despite experiencing severe pain associated with menstruation. In a previous Chinese survey in which women were also classified into three groups according to dysmenorrhea severity, only 7.7% of the patients in the severe group received medical care [14]. Although the consultation rate of this Japanese survey was higher than that in the previous study [14], many Japanese women who wanted to seek medical care for their dysmenorrhea symptoms experienced difficulty and were hesitant to use medical facilities because many people think it is normal to have pain and going to the hospital is unusual [36, 42]. This finding may highlight an aspect of low environmental QOL. Note that the social QOL items comprised three items, while the other subscales consisted of six to eight items. Therefore, it might be possible that, although QOL scores tended to decrease as dysmenorrhea severity increased, social QOL was not significantly affected.

This study highlighted that females in Japan with severe dysmenorrhea have lower psychological QOL scores. In step, the group with severe dysmenorrhea had stronger negative affect during the premenstrual, menstrual, and postmenstrual phases of the MDQ and lower subjective well-being. Moreover, their affective pain was significantly higher—although it is easy to imagine that the greater the severity of dysmenorrhea, the more intense the sensory pain on the SF-MPQ. Dysmenorrhea has been called a chronic form of pain [43] because it can be experienced repeatedly [44]. Chronic pain has been characterized by a fear avoidance model in which a vicious cycle occurs between pain experience, catastrophizing, pain-related fear, avoidant hypervigilance, depression, and an enhanced pain experience [45]. The catastrophizing in this cycle is strengthened by negative emotions [45], which can lead to more affective pain and a lower psychological QOL. Japanese women have been reported to have more negative feelings during menstruation than women in other countries [46, 47], and the Japanese group with severe dysmenorrhea in this study experienced a negative spiral of chronic pain that significantly impacted their QOL. It is helpful to note here that patients with endometriosis are known to have symptoms such as menstrual irregularities, chronic or painful intercourse, and infertility, which often affect their psychological and social functioning [48–50]. Although this study focuses on participants who appear to have no organic disease, it is important to know that similar psychological dysfunction could occur in organic dysmenorrhea with an apparent disease such as endometriosis.

In this study, the percentages of respondents who implemented coping strategies such as taking medications, undergoing medical consultations, and consuming hot drinks was greater in the group with more severe dysmenorrhea. However, there were no significant differences in the percentage of respondents who undertook coping strategies related to stretching and physical activity across severity levels. A previous study indicated the possibility that regular habitual exercise was a useful coping strategy for dysmenorrhea [51]. Considering that the more severe the dysmenorrhea, the more intense the sensory as well as affective pain, exercise could be beneficial in reducing monthly menstrual pain and associated negative emotions [51, 52], thereby improving QOL.

In summary, we considered why the severity of dysmenorrhea was associated with lower QOL in terms of physical and mental QOL and considered coping strategies across different levels of dysmenorrhea severity. The strength of this study is that it is the first to examine the relationship between dysmenorrhea and QOL in a group of Japanese participants. In addition, its detailed assessment of the sensory and affective characteristics of pain and assessment of negative emotions using multiple indicators allowed for a deeper consideration of the causes of reduced QOL in those with severe dysmenorrhea. However, this study had some limitations. First, participants in this study were not diagnosed with primary dysmenorrhea by a medical doctor. There may be a possibility that some of the patients had hidden diseases; however, this cannot be confirmed. Gynecological conditions characterized by chronic pelvic pain may involve significantly higher postoperative pain than other conditions, especially if the diagnosis of endometriosis is delayed, and pain may change from nociceptive to neuropathic [53, 54]. Therefore, early consultation on the presence of organic disease is recommended. Second, this study was a cross-sectional survey. The respondents were asked to respond regardless of their menstrual cycles, and the results might have been affected by the time of the month the survey was taken. Third, recall bias was a possibility because this study was a retrospective survey. Finally, it is possible that questionnaire bias occurred [55]. Nevertheless, the questionnaire took about 10 minutes to complete, and the questions were clearly worded.

This study found clear evidence that young and adult females with severe dysmenorrhea experience a relatively low QOL in Japan. We will use this data as basic information for designing improvements in the system, such as establishing new treatment methods for managing the symptoms of dysmenorrhea, facilitating access to medical attention, and promoting women’s participation in society.

## Supporting information

S1 Checklist (DOCX)

S1 Table Data for analysis. (XLSX)
