# Supplementary material for: Quality of life by dysmenorrhea severity in young and adult Japanese females: A web-based cross-sectional study
Source: PLoS One. 2023 Mar 16;18(3):e0283130. doi: 10.1371/journal.pone.0283130 (PMC10019623; doi:10.1371/journal.pone.0283130)
Supplement: S1 Checklist — (DOCX) [file pone.0283130.s001.docx]

STROBE Statement—checklist of items that should be included in reports of observational studies

|  | Item No. | Recommendation | Page  No. | Relevant text from manuscript |
| --- | --- | --- | --- | --- |
| **Title and abstract** | 1 | (*a*) Indicate the study’s design with a commonly used term in the title or the abstract | 1 | Quality of life by the severity of dysmenorrhea in young Japanese women: A web-based cross-sectional study |
|  |  | (*b*) Provide in the abstract an informative and balanced summary of what was done and what was found | 2-3 | Dysmenorrhea can affect work and schoolwork due to pain and discomfort. However, it is unclear whether the severity of dysmenorrhea in young Japanese women has an impact on their quality of life. The purpose of this study was to compare various factors such as emotions and coping with symptoms according to the severity of dysmenorrhea and determine whether the severity of dysmenorrhea ultimately affects the quality of life.  This web-based cross-sectional survey was sent to 1000 Japanese women aged 16-30. The severity of dysmenorrhea was classified using the Numerical Rating Scale as mild (1-3), moderate (4-7), severe (8-10), and the World Health Organization /Quality of Life- 26 was used as the index of quality of life. One-way ANOVA and Kruskal-Wallis test were used to compare the quality-of-life scores of the groups by dysmenorrhea severity, depending on normality.  First, respondents were divided into two groups: those without dysmenorrhea (n=24) and those with dysmenorrhea (n=471). In the final analysis, respondents of dysmenorrhea classified 156 as mild, 249 as moderate, and 66 as severe, depending on the severity of the menstrual pain. The main findings were that the group with more severe dysmenorrhea had lower quality of life scores, and there were significant differences between groups in the total quality-of-life scores and in the physical, psychological, and environmental subscales.  This study was the first to show the relationship between dysmenorrhea severity and quality of life in Japanese women. In particular, the low psychological quality of life in the severe group of dysmenorrhea was an important point to consider in the room for improvement of dysmenorrhea. This study’s findings were significant for the need to address coping with dysmenorrhea in the future. |
| Introduction | | | |  |
| Background/rationale | 2 | Explain the scientific background and rationale for the investigation being reported | 3-4 | Primary dysmenorrhea, unique to women, is defined as menstrual pain in the absence of pelvic disease, with the main symptom being lower abdominal pain [1]. The pain appears along with the onset of menstruation, is most painful in the first 24-36 hours, and lasts for 2-3 days [2]. A study reported the highest prevalence of dysmenorrhea to be 90% [3], with most women experiencing symptoms approximately once a month [4]. Although the severity of dysmenorrhea varies among individuals [5], dysmenorrhea remains one of the most important health problems that the majority of young women experience.  Previous studies have shown that dysmenorrhea leads to poor academic performance and work efficiency [6, 7], and reduces quality of life (QOL) [8, 9]. Additionally, a survey that calculated labor losses, hospital visits, and medication costs due to dysmenorrhea estimated the annual socioeconomic loss in Japan to be 682.8 billion yen [10]. Investigating the impact of dysmenorrhea and the severity of symptoms is important, not only to improve women's health, but to ensure their QOL. Although there is a movement to encourage women to join the workforce in many countries around the world, including Japan [11], dysmenorrhea may be an obstacle to this movement [6, 7]. However, reports on the relationship between dysmenorrhea and QOL have indicated that the relationship cannot be generalized to other countries [8], and the impact of dysmenorrhea severity on QOL among young women in Japan has not been clarified. |
| Objectives | 3 | State specific objectives, including any prespecified hypotheses | 4 | The purpose of this study was to compare various factors, such as emotions and methods of coping with symptoms, by severity of dysmenorrhea, and ultimately to explore whether the severity of dysmenorrhea affects the QOL of young women. The hypothesis is that greater severity of dysmenorrhea is associated with lower QOL. This study’s significance is in the provision of basic information on the QOL and other aspects of dysmenorrhea in Japanese women by severity of the disease, and to promote the importance of coping with dysmenorrhea. |
| Methods | | | |  |
| Study design | 4 | Present key elements of study design early in the paper | 4 | This study’s anonymous online survey |
| Setting | 5 | Describe the setting, locations, and relevant dates, including periods of recruitment, exposure, follow-up, and data collection | 4 | This study’s anonymous online survey was recruited and conducted from May 17, 2021 to July 7, 2021 by using Google Forms (Alphabet, Mountain View, CA, USA). |
| Participants | 6 | (*a*) *Cohort study*—Give the eligibility criteria, and the sources and methods of selection of participants. Describe methods of follow-up  *Case-control study*—Give the eligibility criteria, and the sources and methods of case ascertainment and control selection. Give the rationale for the choice of cases and controls  *Cross-sectional study*—Give the eligibility criteria, and the sources and methods of selection of participants | 5 | The inclusion criteria were as follows: 1) Japanese women aged 16–30 years old, 2) resident in Japan during the survey period, and 3) agreed to participate in this study. The exclusion criteria were as follows: 1) current or previous history of gynecological or psychiatric disorders. 2) having a severe condition such as heart or malignant disease. |
|  |  | (*b*) *Cohort study*—For matched studies, give matching criteria and number of exposed and unexposed  *Case-control study*—For matched studies, give matching criteria and the number of controls per case | Not applicable |  |
| Variables | 7 | Clearly define all outcomes, exposures, predictors, potential confounders, and effect modifiers. Give diagnostic criteria, if applicable | 6-9 | (The following sections)  2. Survey items and data collection  2.1. Sociodemographic data and basic menstrual information  2.2. Lifestyle  2.3. Intensity of pain associated with menstruation  2.4. Negative emotions and well-being  2.5. Coping strategies for symptoms of dysmenorrhea  2.6. QOL |
| Data sources/ measurement | 8* | For each variable of interest, give sources of data and details of methods of assessment (measurement). Describe comparability of assessment methods if there is more than one group | 6-9 | (The following sections)  2. Survey items and data collection  2.1. Sociodemographic data and basic menstrual information  2.2. Lifestyle  2.3. Intensity of pain associated with menstruation  2.4. Negative emotions and well-being  2.5. Coping strategies for symptoms of dysmenorrhea  2.6. QOL |
| Bias | 9 | Describe any efforts to address potential sources of bias | 6 | The following efforts were made to reduce bias in this study: 1) The survey was conducted at other sites (12 universities and 10 companies) rather than at a single site to avoid selection bias, 2) Subject information was anonymized, 3) Because it was a web-based questionnaire, the researcher and the subjects were not acquainted with each other, 4) The subjects were grouped according to the results of the questionnaire, and they did not know which group they would belong to when they responded to the questionnaire. |
| Study size | 10 | Explain how the study size was arrived at | 8-9 | As there are no reports on the relationship between the degree of dysmenorrhea and QOL in Japanese women, the amount of effect size expected when comparing the main outcome, defined as QOL, among the three groups was not known. Therefore, we calculated the sample size using an effect size of 0.25, an alpha error of 0.05, and a mean power of 0.80 by G*power (version 3.1.9.2, Heinrich-Heine-University Düsseldorf, Düsseldorf, Germany) [30]. From these calculations, we estimated that a total of 159 respondents were required for the three groups with 53 respondents per group. |

Continued on next page

| Quantitative variables | 11 | Explain how quantitative variables were handled in the analyses. If applicable, describe which groupings were chosen and why | 6-8 | Page 6: To assess the amount of physical activity, the International Physical Activity Questionnaire–Short Form [14, 15] was used. Total physical activity was calculated by the weekly average amount of walking, moderate physical activity, and vigorous physical activity (Metabolic equivalent tasks [METs]*mins/week). One Met is approximately equal to the energy consumption required for a person to sit quietly. The amount of physical activity was also classified into three levels: low (<600 METs*mins/week), moderate (600–2999 METs*mins/week), and high (≥3000 METs*mins/week) [16].  Page 7: We classified scores of 1 to 3 as mild dysmenorrhea, scores of 4 to 7 as moderate dysmenorrhea, and scores of 8 to 10 as severe dysmenorrhea [9, 21].  Page 7: A supplementary evaluation of menstrual pain, the short-form McGill pain questionnaire (SF-MPQ), was also used [22, 23]. The SF-MP is useful for analyzing qualitative, not only quantitative aspects of pain, and consists of 15 pain descriptions, 11 sensory and 4 affective. Each question was on a 0 to 3-point Likert scale, with higher scores indicating stronger levels of pain.  Page 7-8: The Menstrual Distress Questionnaire (MDQ) is a commonly used measurement scale to assess the severity of symptoms related to menstruation [24]. We used the subscale of “negative affect” to evaluate the negative psychological aspects of premenstrual, during menstrual, and postmenstrual periods. Responses were scored using a 6-point Likert scale, ranging from 1 (no reaction at all) to 6 (acute or partially disabling), with higher scores indicating a greater severity of mental symptoms.  Page 8: To assess well-being and mental health, the Japanese version of the WHO five well-being index (WHO-5-J) was used [25]. The WHO-5-J consists of a total of five questions, and responses to each item were rated on a six-point Likert scale from 0 to 5. The maximum score is 25 points, with higher scores indicating better well-being.  Page 9: Respondents were divided into mild, moderate, and severe dysmenorrhea depending on the intensity of menstrual pain. |
| --- | --- | --- | --- | --- |
| Statistical methods | 12 | (*a*) Describe all statistical methods, including those used to control for confounding | 9 | A descriptive analysis was performed, with quantitative variables expressed as means and standard deviations, and qualitative variables examined as frequencies and percentages. The one-way analysis of variance was used to compare height, well-being, and QOL (physical, psychological, environmental) between the 3 groups to confirm normality by the Shapiro-Wilk test. The Kruskal-Wallis test and Bonferroni post-hoc tests were conducted to compare other scores of continuous variables such as sociodemographic data and basic menstrual information, the intensity of pain associated with menstruation, negative emotions and well-being, and QOL. The chi-square test was used to compare differences in the distribution of basic menstrual information, lifestyle, and coping strategies across the 3 groups. All the data were analysed using IBM SPSS Statistics for Windows (version 23.0; IBM Corp., Armonk, NY, USA). |
|  |  | (*b*) Describe any methods used to examine subgroups and interactions | Not　applicable |  |
|  |  | (*c*) Explain how missing data were addressed | 9 | All subjects who missed answers or chose the "do not want to answer" option were excluded. |
|  |  | (*d*) *Cohort study*—If applicable, explain how loss to follow-up was addressed  *Case-control study*—If applicable, explain how matching of cases and controls was addressed  *Cross-sectional study*—If applicable, describe analytical methods taking account of sampling strategy | 9 | As there are no reports on the relationship between the degree of dysmenorrhea and QOL in Japanese women, the amount of effect size expected when comparing the main outcome, defined as QOL, among the three groups was not known. Therefore, we calculated the sample size using an effect size of 0.25, an alpha error of 0.05, and a mean power of 0.80 by G*power (version 3.1.9.2, Heinrich-Heine-University Düsseldorf, Düsseldorf, Germany) [30]. From these calculations, we estimated that a total of 159 respondents were required for the three groups with 53 respondents per group. |
|  |  | (*e*) Describe any sensitivity analyses | Not　applicable |  |
| Results | | | | |
| Participants | 13* | (a) Report numbers of individuals at each stage of study—eg numbers potentially eligible, examined for eligibility, confirmed eligible, included in the study, completing follow-up, and analysed | 10 | Of the 1000 distribution targets, we received answers from a total of 717 respondents (71.7%). Of these respondents, 57 had a current and previous history of gynecological or psychiatric disorders, and 165 responses were incomplete with some respondents being unwilling to answer certain questions. Therefore, 495 respondents (49.5%) were included in the current analysis. |
|  |  | (b) Give reasons for non-participation at each stage | 10 | Of these respondents, 57 had a current and previous history of gynecological or psychiatric disorders, and 165 responses were incomplete with some respondents being unwilling to answer certain questions. |
|  |  | (c) Consider use of a flow diagram | Figure 1 |  |
| Descriptive data | 14* | (a) Give characteristics of study participants (eg demographic, clinical, social) and information on exposures and potential confounders | 10, Table 1 | Table 1 shows the basic information and basic menstrual data of dysmenorrhea respondents in this study. The mean ages of the mild, moderate, and severe groups were 22.8 years (SD=3.2), 22.4 years (SD=3.2), and 22.3 years (SD=2.8), respectively (p=0.026). There were statistically significant differences in the duration of pain, amount of menstruation, and family history of dysmenorrhea among the three groups (p<0.05, respectively). There were no statistically significant differences among the three groups in terms of weight, height, BMI, menarche age, menstrual cycle, and physical activity level. |
|  |  | (b) Indicate number of participants with missing data for each variable of interest | 10 | 165 responses were incomplete with some respondents being unwilling to answer certain questions. |
|  |  | (c) *Cohort study*—Summarise follow-up time (eg, average and total amount) | Not　applicable |  |
| Outcome data | 15* | *Cohort study*—Report numbers of outcome events or summary measures over time | Not　applicable |  |
|  |  | *Case-control study—*Report numbers in each exposure category, or summary measures of exposure | Not　applicable |  |
|  |  | *Cross-sectional study—*Report numbers of outcome events or summary measures | Table 2, Table 3, Table 4,  Figure 2,  Figure 3 |  |
| Main results | 16 | (*a*) Give unadjusted estimates and, if applicable, confounder-adjusted estimates and their precision (eg, 95% confidence interval). Make clear which confounders were adjusted for and why they were included | Not　applicable |  |
|  |  | (*b*) Report category boundaries when continuous variables were categorized | Table 2, Table 3, Table 4,  Figure 2, Figure 3 |  |
|  |  | (*c*) If relevant, consider translating estimates of relative risk into absolute risk for a meaningful time period | Not　applicable |  |

Continued on next page

| Other analyses | 17 | Report other analyses done—eg analyses of subgroups and interactions, and sensitivity analyses | Not　applicable |  |
| --- | --- | --- | --- | --- |
| Discussion | | | | |
| Key results | 18 | Summarise key results with reference to study objectives | 19 | This study aimed to explore whether the severity of dysmenorrhea affects the QOL of young women. The main results were that the more severe dysmenorrhea group had lower total QOL scores and physical, psychological, and environmental QOL by subscale, with significant differences in QOL scores among groups by severity of dysmenorrhea. This is the first study to show that the group with more severe dysmenorrhea has a lower QOL in Japanese women. |
| Limitations | 19 | Discuss limitations of the study, taking into account sources of potential bias or imprecision. Discuss both direction and magnitude of any potential bias | 21 | There are limitations to this study. First, this study was a cross-sectional survey. The respondents were asked to respond regardless of their menstrual cycle. The results might have been affected by the time of the month the survey was taken. Second, recall bias was a possibility because this study was a retrospective survey. Third, it is possible that questionnaire bias occurred [38]. Nevertheless, the questionnaire took about 10 minutes to complete, and the questions were clearly worded. |
| Interpretation | 20 | Give a cautious overall interpretation of results considering objectives, limitations, multiplicity of analyses, results from similar studies, and other relevant evidence | 19-21 | The physical aspects of WHO/QOL-26 included items that asked about the degree to which physical pain or discomfort limits daily activities. It was known from previous studies in other countries that dysmenorrhea affects performance at school and work [6, 7]. It was meaningful that we found that the more severe the dysmenorrhea, the more likely the performance of Japanese women was affected. The environmental QOL included questions such as the degree of healthfulness of living environment and satisfaction with the accessibility of health care facilities and social services. Only 21.2% of the severe group in this study had seen a medical provider despite severe pain associated with menstruation. In a previous Chinese survey, women were classified into three groups according to the severity of dysmenorrhea, and only 7.7% of the patients in the severe group received medical care [9]. Although the consultation rate of this Japanese survey was higher than that in the previous study [9], many Japanese women who wanted to seek medical care regarding symptoms of dysmenorrhea felt difficulty and hesitated to use medical facilities because many people think it is normal to have pain, and going to the hospital is unusual [26, 31]. This could indicate an aspect of low environmental QOL. Note that the social QOL items comprised three items, while the other subscales consisted of six to eight items. Therefore, it might be possible that although there was a trend toward lower QOL scores with greater severity of dysmenorrhea, social QOL was not detected as a significant difference.  This study provided a view related to lower psychological QOL scores in the severe group of dysmenorrhea. Complementing this, the group with severe dysmenorrhea had stronger negative affect during the premenstrual, menstrual, and postmenstrual phases of the MDQ and lower subjective well-being. It was also important to reveal that affective pain was significantly higher, although it was easy to imagine that the greater the severity of dysmenorrhea by definition of the grouping, the more intense the sensory pain was on the SF-MPQ. Dysmenorrhea has been noted to have an aspect of chronic pain [32], because women experience the pain repeatedly [4]. Chronic pain has been characterized by a fear avoidance model in which a vicious cycle occurs of pain experience, catastrophizing, pain related fear, avoidance hypervigilance, depression, and enhanced pain experience [33]. The catastrophizing in this cycle is strengthened by negative emotions [33], with the possibility of leading to further affective pain and reduction of psychological QOL. Japanese women have been reported to have more negative feelings during menstruation than women in other countries [34, 35], and the Japanese group with severe dysmenorrhea in this study was found to experience a negative spiral of chronic pain with a significant impact on their QOL.  In this study, the percentage of respondents who implemented coping strategies such as taking medications, undergoing medical consultations, and consuming hot drinks were larger in the group with more severe symptoms of dysmenorrhea. However, there was no significant difference in the percentage of respondents who undertook coping strategies at any severity level for stretching and physical activity exercises. A previous study indicated the possibility that regular habitual exercise was a useful coping strategy for dysmenorrhea [36]. Considering that the more severe the dysmenorrhea, the more intense the sensory as well as the affective pain, exercise could be beneficial in reducing monthly menstrual pain, and associated negative emotions [36, 37], thereby improving QOL. |
| Generalisability | 21 | Discuss the generalisability (external validity) of the study results | 19 | It was known from previous studies in other countries that dysmenorrhea affects performance at school and work [6, 7]. It was meaningful that we found that the more severe the dysmenorrhea, the more likely the performance of Japanese women was affected. |
| Other information | |  | | |
| Funding | 22 | Give the source of funding and the role of the funders for the present study and, if applicable, for the original study on which the present article is based | Not　applicable |  |

*Give information separately for cases and controls in case-control studies and, if applicable, for exposed and unexposed groups in cohort and cross-sectional studies.

**Note:** An Explanation and Elaboration article discusses each checklist item and gives methodological background and published examples of transparent reporting. The STROBE checklist is best used in conjunction with this article (freely available on the Web sites of PLoS Medicine at http://www.plosmedicine.org/, Annals of Internal Medicine at http://www.annals.org/, and Epidemiology at http://www.epidem.com/). Information on the STROBE Initiative is available at www.strobe-statement.org.
